# Isolation and Characterization of Novel Huperzine-Producing Endophytic Fungi from *Lycopodiaceae* Species

**DOI:** 10.3390/jof9121134

**Published:** 2023-11-24

**Authors:** Thanh Thi Minh Le, Ha Thanh Pham, Ha Thi Thu Trinh, Hoa Thi Tran, Ha Hoang Chu

**Affiliations:** 1 Institute of Biotechnology, Vietnam Academy of Science and Technology, Hanoi 100000, Vietnam; 2 Vietnam Academy of Science and Technology, Graduate University of Science and Technology, Hanoi 100000, Vietnam

**Keywords:** Alzheimer, endophytic fungi, huperzine A, huperzine B, *Lycopodium clavatum*, *Phlegmariurus phlegmaria*, *Phlegmariurus squarrosus*

## Abstract

Huperzine A (HupA) is an important drug for treating Alzheimer’s disease (AD) and is primarily extracted from the *Huperzia serrata* (*Lycopodiaceae*). Failures in the chemical synthesis of Hup and in vitro culture have put *H. serrata* in danger of extinction, and there is a need for an extensive investigation of Hup from alternative perspectives. The aim of this study is to identify endophytic fungi that produce high Hup or simultaneously produce many types of Hup and have high genetic stability derived from other *Lycopodiaceae* species as a source of materials for natural Hup production. In this work, Hup-producing endophytic fungi were isolated from three species: *Lycopodium clavatum*, *Phlegmariurus squarrosus,* and *P. phlegmaria*. Of these, *L. clavatum* and *P. squarrosus* were confirmed as novel sources of Hup-producing fungi. Based on morphological characteristics and nuclear ribosomal DNA ITS sequences, four endophytic fungi *Colletotrichum siamense* THG1-17, *Epicoccum sorghinum* THG01-18, *Phoma* sp. TKH3-2, and *Phyllosticta* sp. THG2-27 were firstly isolated from these *Lycopodiaceae* plants, which were capable of simultaneously producing both HupA and HupB, as evidenced by high-performance liquid chromatography analysis. The four strains showed stability in Hup yield over 50 generations of culture with an in vitro storage period of 3 months. These isolated fungi will provide a new source of materials for further research to develop drugs containing HupA as well as HupB for AD treatment in the future.

## 1. Introduction

Alzheimer’s disease (AD) is a progressive disease that destroys memory and other important mental functions due to reduced levels of the neurotransmitter acetylcholine (ACh) in the cerebral cortex, causing memory impairment [1]. Although AD has been defined for about 100 years, its molecular mechanisms and pathogenesis are still not fully understood [2]. A lot of effort has been made to find new drugs targeting AD through new mechanisms [3]. However, acetylcholinesterase (AChE) inhibitors that block cholinergic degeneration of ACh are considered a promising approach in the treatment of AD and are currently approved by the FDA for use in AD to delay the progression of the disease [4].

Huperzine A (HupA) and huperzine B (HupB) are Lycopodium alkaloids capable of selectively inhibiting AChE activity with unique pharmacological properties and devoid of unexpected toxicity associated with other chemotherapeutic drugs (tacrine, rivastigmine, galanthamine, and donepezil) [5,6,7]. The potency of the AChE inhibition (AChEI) of HupA is similar or even superior, with fewer undesired effects compared to other pharmaceutical drugs [8,9]. HupB demonstrates lower AChEI activity than HupA but exhibits a higher therapeutic index and lower toxicity [10,11]. HupA and HupB act directly on the brain at low doses (dosage in micrograms) by crossing the blood–brain barrier and inhibiting the production of AChE. Inhibition of AChE leads to increasing the concentration of acetylcholine in the brain, improving cognition and memory for Alzheimer’s patients [12,13,14]. Huperzines have also been proven to not only support the brains of Alzheimer’s patients and elderly people with memory impairment but also to effectively improve the memory of healthy adults. Thus, HupA and HupB have been considered medications, improving the levels of neurotransmitters in the brain.

The *Lycopodiaceae* species are known as valuable medicinal herbs for treating dementia and are found in some countries of Asia, North America, and Europe [15,16,17,18,19,20]. Huperzines have been determined in more than 50 *Lycopodiaceae* species from Australia, French Polynesia, Panama, Mexico, Malaysia, Poland, and, particularly, China, where *Lycopodiaceae* is distributed widely. Most of them produce low HupA concentrations, and the level of HupB concentration has been rarely quantified [15,16,17,18,19,20,21]. However, the content of these huperzines in plants is low, and overexploitation has reduced the medicinal plant population in the wild [6,15,21]. There have been some studies on in vitro culture *Huperzia serrata* and *H. selago* to obtain huperzine, but the efficiency of this method has not been high due to strict culture conditions, a long incubation time, and a low yield [22,23]. The chemical synthesizing method also generates a large amount of huperzines, but it is not a feasible method because of the complicated technique, high production cost, and many other derivatives leading to unwanted effects compared with natural huperzines [24,25,26].

The endophytic fungi associated with *Lycopodiaceae* plants have the ability to produce HupA with high concentration. The microbial source of huperzine is available, and the Hup-producing microorganisms can open up a promising approach for producing drugs containing huperzine to treat AD [27]. So far, some endophytic HupA-producing fungi associated with *Lycopodiaceae* plants such as *H. serrata*, *Phlegmariurus phlegmaria*, and *P. taxifolius* were identified and determined the ability to produce HupA, including genera: *Aspergillus*, *Trichoderma*, *Alternaria*, *Paecilomyces*, *Podospora*, *Penicillium*, *Mucor*, *Cyphellophora*, *Colletotrichum*, *Hypoxylon*, *Acremonium*, *Blastomyces*, *Schizophyllum*, and *Botrytis* [27,28,29,30,31,32]. Some of them showed high HupA production; for example, *Trichoderma harzianum* NSW-V (*H. serrata*) had the highest hupA content of 319.8 mg L^−1^ liquid culture [27]. However, the studies on Hup-producing endophytic fungi from other *Lycopodiaceae* species remain limited. It is necessary to find endophytic fungi that produce high Hup or simultaneously produce many types of Hup as a source of materials for the production of natural Hup. On the other hand, these fungi are very susceptible to mutation and lose the original expressing ability to produce Hup. Therefore, studying the genetic stability in the Hup biosynthesis process of fungal strains is necessary in order to use them as a source of materials in industrial-scale production in the future. Our previous studies reported that *Penicillium* sp. LDL4.4 and *Fusarium* sp. Rsp5.2 recovered from *H. serrata* were producers of HupA [33,34]. The present study was a follow-up to our previous studies and the first report to prove and shed light on novel endophytic fungi associated with the three plants (*L. clavatum*, *P. phlegmaria*, and *P. squarrosus*) which are capable of stably and potentially producing both HupA and HupB as promising sources of materials for further research in supporting the treatment of AD in the future.

## 2. Materials and Methods

### 2.1. Plant Materials

The wild samples of *Lycopodiaceae* plants were collected in May 2020 from the natural populations in the tropical mountains of the northeast province (Ha Giang) and south-central province (Khanh Hoa) of Vietnam. Plants were identified, deposited at the National Institute of Medicinal Materials, and confirmed for the presence of HupA and HupB depending on each species (see Table 1).

### 2.2. Isolation of Endophytic Fungi

Healthy plant samples of *Lycopodiaceae* were surface sterilized, as described previously [33]. The samples were rinsed under running tap water, and then the stems, leaves, and roots were separated. They were sterilized sequentially by washing for 5 min in 75% ethanol, 5 min in 10% sodium hypochlorite, and 2 min with 0.1% mercuric chloride. Finally, the samples were rinsed in sterile distilled water three times. The last rinsing water was spread on the potato dextrose agar (PDA-HiMedia, Mumbai, India) plates and incubated at 28 °C in the dark for 7 days as controls to ensure that the surface sterilization had eliminated all epiphytic microorganisms adhering to the segments externally. Each sample of the stems, roots, and leaves was cut into small pieces (0.2 to 0.5 cm) and placed about 1.5 to 2 cm apart in 9 cm diameter petri dishes containing PDA with antibiotic streptomycin (50 µg mL^−1^) and penicillin (100 µg mL^−1^) to prevent any bacterial growth. The plates were incubated at 28 °C in the dark and checked daily for any fungal growth. Individual hyphal tips of fungal colonies were carefully placed onto new PDA plates to obtain pure isolates. The pure cultures were preserved in PDA slant tubes for storing at 4 °C and as mycelia with spores in 15% glycerol at −20 °C. All fungal strains were deposited at the VAST-Culture Collection of Microorganisms (VCCM), Institute of Biotechnology, Vietnam Academy of Science and Technology.

### 2.3. Identification of Endophytic Fungi

The fungal strains were morphologically identified on PDA plates under ambient daylight conditions at 28 °C. Hyphae, sporulation, and conidia were observed by light microscopy (Olympus, Tokyo, Japan) following the previous procedure [33,34].

For molecular identification based on the Internal Transcribed Region (ITS) sequence analyses, the strain was inoculated into 250 mL Erlenmeyer flasks containing 50 mL potato dextrose broth (PDB-HiMedia, Mumbai, India) medium and cultured at 150 rpm on a rotary shaker at 28 °C for 3 to 10 days depending on each strain. The genomic DNA was extracted using a QIAamp DNA mini Kit (Qiagen, Hilden, Germany) according to the manufacturer’s instructions. For amplification of (ITS sequences, the universal primers ITS1 (5′-CCGTAGGTGAACCTGCGG-3′), and ITS4 (5′-CCTCCGCTTATTGATATGC-3′) were employed to obtain ~500-bp PCR product. The resulting PCR products were examined by electrophoresis in 0.8% agarose gels in TAE buffer and then purified using a QIAquick PCR Purification Kit (Qiagen, Hilden, Germany). The PCR products were sequenced using an AEI PRISM@ 3700 Genetic Analyzer (Thermo Fisher Scientific, Waltham, MA, USA). The ITS sequences were trimmed and compared with ITS sequences available in GenBank databases using the BLASTn program (NCBI—National Center for Biotechnology Information, Bethesda, MD, USA). The ITS sequences were deposited onto the GenBank.

### 2.4. Preparation of Fungal Extracts

The fungal strain was cultured in 1000 mL PDB medium and shaken at 150 rpm at 28 °C for four to 7 days. The mycelia was harvested by centrifugation at 6000 rpm for 10 min, dried at 45 °C overnight, and ground to powder using a cutter mill (Philips, Tokyo, Japan).

The extraction of Hup was, according to Zhu et al. (2010), with modifications [33,35]. The powders were weighed accurately for each sample. The powder sample was moistened with 10% ammonia (*w*/*v*, 1:1) for 30 min and then extracted by shaking with a ratio of 30:1 of 5% hydrochloric acid for 16 h. After the elimination of dregs by filtering, 25% ammonia solution (pH 9.0) was added to the water phase, followed by extraction 3 times with chloroform with a ratio of 1:1. Finally, the combined extracts were evaporated to dryness by a rotary evaporator at approximately 40–45 °C. The obtained dry residue was dissolved in 1 mL methanol.

### 2.5. HPLC-DAD-MS Analysis

The crude and purified extracts containing HupA and HupB were detected using HPLC-DAD-MS. The HPLC analysis was performed on a Thermo Dionex Ultimate 3000 system (Thermo Fisher Scientific, Waltham, MA, USA) with a diode-array detector (DAD) monitored from 200 to 500 nm, which was coupled to an LCQ Fleet Ion Trap MS spectrometer (Thermo Fisher Scientific, Waltham, MA, USA). The separation of HupA and HupB was conducted with a Hypersil Gold C18 column (250 × 4.6 mm, 5 μm) thermostated at 40 °C. The indicator solution and the analyzed samples were filtered through a 0.45 μm filter before injection. Five microliters of the methanolic extracts were injected. A solution of water (0.1% formic acid) and acetonitrile were set as solvent channels A and B, respectively, at a flow rate of 0.4 mL min^−1^ with 0.1% formic acid gradient in the 80–0% range and acetonitrile gradient in the 20–100% range. The UV detector was set to 310 nm.

Quantification was achieved using the standard curve generated from the HupA and HupB standards (Sigma-Aldrich, Mo., USA) over a 0.01–0.5 mg mL^−1^ concentration range at which the peak area and height exhibited linear relationships with the absorbance (HupA = r^2^ = 0.99996, HupB = r^2^ = 0.9997).

Electrospray ionization mass spectra (ESI-MS) were used to confirm the ion [M+H]^+^ of HupA and B in the chromatograms. Mass spectroscopy was performed on purified Hup samples. The electrospray ionization combined with mass spectrometry spectrum was obtained using an Agilent 6120 Single Quadrupole LC–MS Agilent 1260 system (Agilent, Santa Claraa, CA, USA).

### 2.6. AChE Inhibition

AChE inhibitory activity of the purified fungal extracts was determined by Ellman’s method [36]. The reaction mixture included 30 µL of AChE (2 U/mL), 2810 µL of 0.1 M phosphate buffer (pH 8.0), and 30 μL of tested samples followed by incubation at 10 min at 25 °C. After that, 100 μL of 5′dithiobis-2-nitrobenzoic acid (DTNB) (286 μM) and 30 μL of acetylthiocholine iodide (0.86 mM) were added to the mixture and incubated again for 30 min at 25 °C. For the controls, 30 µL of 2 U/mL AChE was replaced with a similar volume of 0.1 M phosphate buffer (pH 8.0). Standard HupA and HupB were used as positive controls and for comparison. The absorbance at 412 nm was recorded using a microwell plate reader Apel PD303 (Apel, Kawaguchi, Saitama, Japan). The IC_50_ was performed with a volume of the test solution at various concentrations (0.5 ÷ 200 μg mL^−1^) for each extract and standard Hup. Percent inhibition was calculated using the following formula: (Absorbance of control-absorbance of sample)/Absorbance of control × 100. As a result, IC_50_ values were calculated using Microsoft Excel and logistic regression analysis of three independent experiments.

### 2.7. Growth Dynamics and Hup Biosynthesis Ability of Endophytic Fungi

A 5 × 5 mm block of mycelium from a PDA culture of Hup-producing endophytic strains was cultured in 250 mL PDB medium and shaken at 150 rpm at 28 °C for 2, 4, 6, 8, 10, and 12 days with three replicates at each time period. The mycelia of three replicates were harvested by centrifugation at 6000 rpm for 10 min, dried at 45 °C overnight, and ground to powder. The extraction and yield of Hup from the strains were determined by the methods mentioned above. The collected data were statistically analyzed using Microsoft Office Excel 2010 software.

### 2.8. Hereditary Stability and Hup Yield of Endophytic Fungi

The genetic stability of the wild strains was studied by continuous culture on PDA medium at 28 °C for 7 days for 1 generation. Isolates of the 10th, 20th, 30th, 40th, and 50th generations were preserved for 3 months in 15% glycerol at 4 °C cold conditions. After 3 months, the isolates were cultured on PDA medium for 7 days and then continued to be grown in PBB medium at 28 °C with shaking at 150 rpm for 8 days for three replicates. The collection of fungal biomass, extraction, determination of Hup A and B contents, and data analysis were carried out according to the above methods.

## 3. Results

### 3.1. Isolation and Morphology of Endophytic Fungi in Lycopodiaceae Species

**Figure 1:**
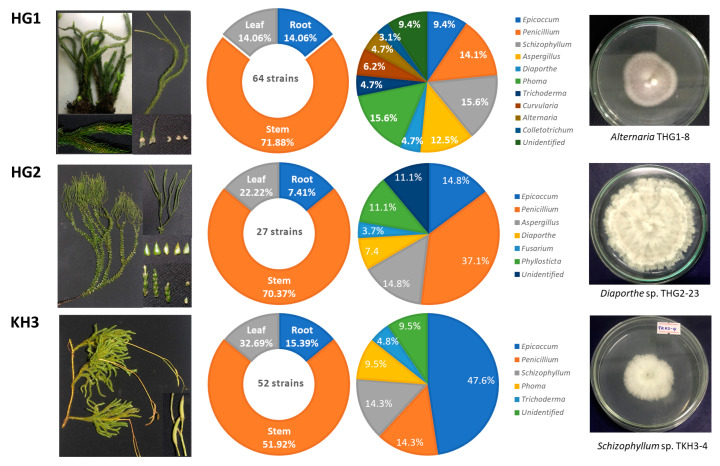
Isolation and distribution of endophytic fungi recovered from three *Lycopodiaceae* species. *P. squarrosus* (HG1), *P. phlegmaria* (HG2), and *L. clavatum* (KH3).

A total of 143 endophytic fungi were isolated from fresh plant samples of three *Lycopodiaceae* species collected from natural populations in the Khanh Hoa and Ha Giang provinces of central and north Vietnam (*P. squarrosus* (HG1)—64 strains, *P. phlegmaria* (HG2)—27 strains, and *L. clavatum* (KH3)—52 strains). Out of all fungal isolates from three plants, the maximum number of isolates were obtained from stems (51.92 ÷ 71.88%), followed by leaves (14.06 ÷ 32.69%), and roots (7.41 ÷ 15.39%) (Figure 1).

The morphology of conidia, colonies, and unique phenotypic characteristics of these isolates were identified. Of the 64 fungal strains from *P. squarrosus*, 58 strains belonged to ten genera, and 6 were unidentified strains (9.4%). The genera *Schizophyllum* and *Phoma* (15.6%), *Penicillium* (14.1%), and *Aspergillus* (12.5%) were the most predominant endophytes. A lower number of fungal strains were recorded for *Epicoccum* (9.4%); *Curvularia* (6.2%); *Diaporthe*, *Alternaria*, and *Trichoderma* (4.7%); and *Colletotrichum* (3.1%) (Figure 1).

From *P. phlegmaria*, the 27 strains were classified into 6 genera of 24 isolates and 3 unidentified strains (11.1%). The genus *Penicillium* (37.1%) was the most frequent endophytic species, followed by other genera *Aspergillus* and *Epicoccum* (14.8%), *Phyllosticta* (11.1%), *Diaporthe* (7.4%), and *Fusarium* (3.7%).

The 52 strains isolated from *L. clavatum* were confirmed to belong to 5 genera, with 47 strains and 5 unidentified strains (9.5%). Among them, the majority genus was *Epicoccum*, with 47.6% (25 strains). The remaining genera showed a much lower number of fungal strains, including genera *Penicillium* and *Schizophyllum* (14.3%), *Phoma* (9.5%), and *Trichoderma* (4.8%) (Figure 1).

The results also showed that two fungal genera, *Penicillium* and *Epicoccum*, were found in all three plant species, while genera *Alternaria*, *Colletotrichum*, *Fusarium*, *Curvularia,* and *Phyllosticta* appeared in only one plant species. Five fungal genera, *Aspergillus*, *Schizophyllum*, *Phoma*, *Trichoderma,* and *Diaporthe*, were isolated from two plants. This shows that the diversity of endophytic fungi is specific to each plant species.

### 3.2. Screening of HupA- and HupB-Producing Fungi Based on Chromatographic Separation

**Figure 2:**
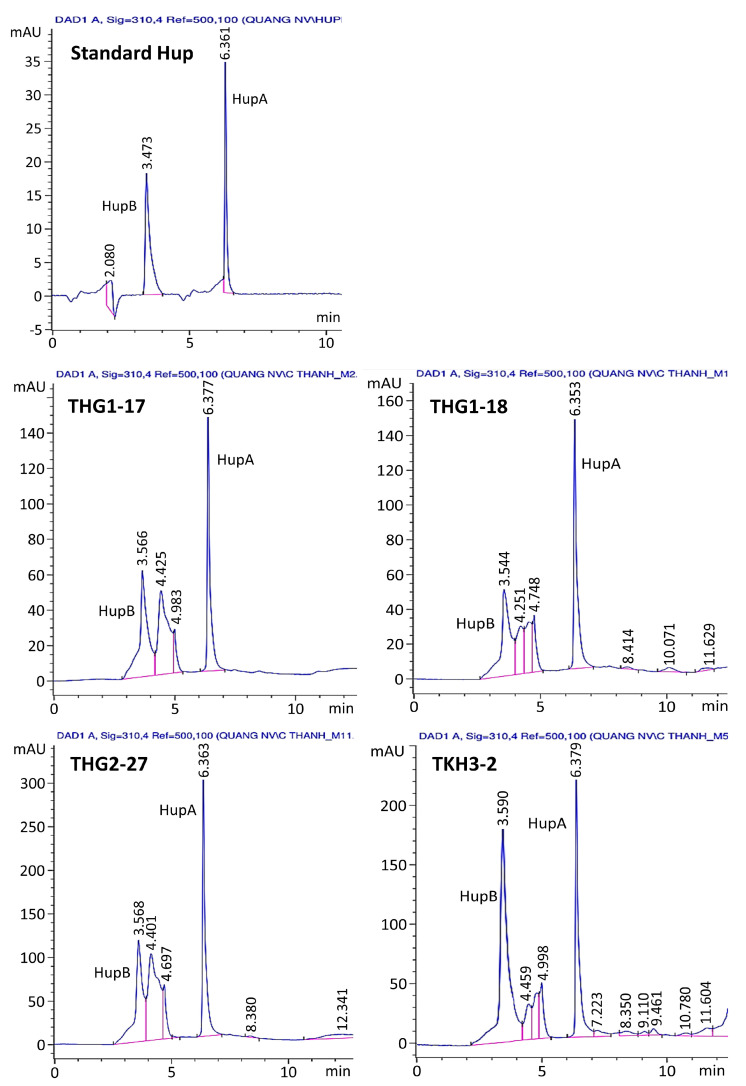
HPLC analysis of standard huperzines and the crude alkaloid extracts of 4 fungal endophytes. The mobile phase was 0.1% formic acid and acetonitrile at a flow rate of 0.4 mL min^−1^ with 0.1% formic acid gradient in the 80–0% range and an acetonitrile gradient in the 20–100% range.

The crude extracts of the 143 fungal strains were examined for the presence of HupA and HupB by HPLC analysis. The identification of HupA and B in extracts from fungi was performed by comparing UV spectra and retention times with values of standards HupA and B (Figure 2). The results of HPLC analysis confirmed the presence of both HupA and B at four strains, including strains THG1-17 and THG-1.18 (*P. squarrosus*), HG2-27 (*P. phlegmaria*), and TKH3-2 (*L. clavatum*). The retention time of HupA from fungal extracts was about 6.353 ÷ 6.379 min, and for HupB, it was about 3.544 ÷ 3.590 min, which was similar to the retention time of standard HupA (6.361 min) and standard HupB (3.473 min) at a 310 nm wavelength (Figure 2).

### 3.3. Quantification and Confirmation of HupA and HupB in Fungi

Four purified extracts containing Hup were quantified for their ability to produce HupA and B using HPLC. An HPLC analysis of the dried mycelium was obtained from 1 L of PBD medium, the contents of HupA were quantifed from 0.147 to 0.324 mg gdcw^−1^, and the contents of HupB were quantifed from 0.106 to 0.263 mg gdcw^−1^ (Table 2). The high HupA-producing strain was THG2-27 (0.324 mg gdcw^−1^), which also had a high HupB concentration for a sample (0.197 mg gdcw^−1^). Another potential high hup-containing strain was the TKH3-2 strain, which produced the highest HupB content of 0.263 mg gdcw^−1^ and potential HupA content of 0.221 mg gdcw^−1^. Strain THG1-18 had the lowest Hup contents (HupA/HupB = 0.147/0.106 mg gdcw^−1^) (Table 2).

The HupA and HupB produced by the fungal strains were further identified by electrospray mass spectroscopy (LC-MS). MS spectra (positive mode) of samples confirmed the ion [M+H]^+^ of HupA at m/z 243 and [M+H]^+^ of HupB at m/z 257 in the mass spectrums, which were relatively similar to the mass spectrums of standard HupA and B.

### 3.4. Identification of Hup-Producing Endophytic Fungi Based on Morphology and ITS Sequences Analysis

**Figure 3:**
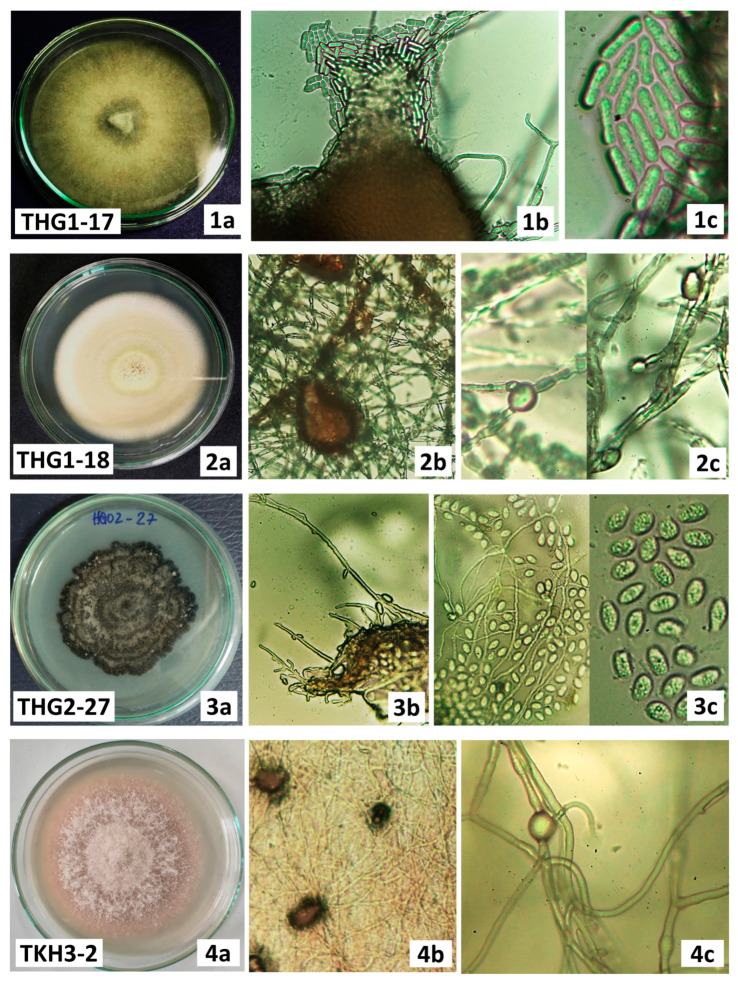
The morphological characteristics of colony (**1a**,**2a**,**3a**,**4a**), pycnidia (**1b**,**2b**,**3b**,**4b**), chlamydospores (**1c**,**2c**), and conidia (**3c**,**4c**) by light microscope (10× and 40×) of Hup-producing fungal strains.

The four Hup-producing endophytic strains were identified by morphology and ITS sequences analysis. The isolates were identified based on the morphology of conidia, colonies, and unique phenotypic characteristics (Figure 3). The ITS regions of the ribosomal DNA (ITS1-5.8S-ITS2) from four strains were amplified by PCR, sequenced, and analyzed by BLAST-searching the GenBank database (Table 3). The size of ITS regions ranged from 482 bp to 602 bp. ITS-based rDNA sequence analysis revealed that all ITS sequences were no less than 99% similar to the closest match from the GenBank database. Four fungal strains belonged to four genera, including *Colletotrichum*, *Epicoccum*, *Phoma*, and *Phyllosticta*, which were in agreement with morphological results (Figure 3). The sequences of two strains, THG1-17 and THG1-18, had maximum sequence identity and query coverage of more than 99% based on data available in GenBank. Combining morphological characteristics, two strains were classified as *Colletotrichum siamense* THG1-17 and *Epicoccum sorghinum* THG01-18. The remaining two strains were identified as belonging to two genera *Phoma* sp. TKH3-2 and *Phyllosticta* sp. THG2-27 when their sequences had maximum sequence similarity of more than 99%, but the query coverage was only less than 95% with reference sequences available onto the GenBank database. All ITS sequences were submitted to GenBank (Table 3).

Four fungal strains were deposited at the VAST-Culture Collection of Microorganisms, Institute of Biotechnology, Vietnam Academy of Science and Technology with accession numbers: VCCM 44310 (THG1-17), VCCM 44311 (THG1-18), VCCM 44312 (THG2-27), VCCM 44314 (TKH3-2).

### 3.5. AChE Inhibition

**Figure 4:**
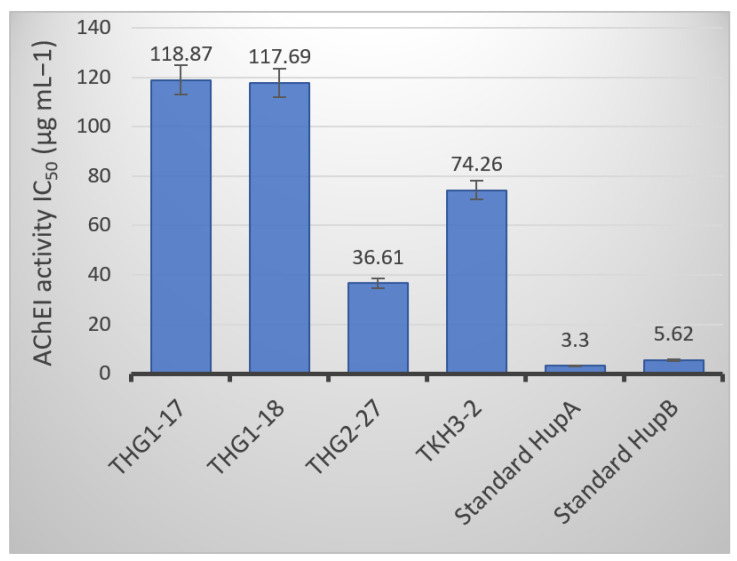
The IC_50_ values of AChE inhibitory activity by the huperzine extracts of endophytic fungi.

The AChE inhibition activity of Hup produced by fungal strains was compared with the AChE inhibition activity of standards HupA and HupB in vitro, which had AChE inhibitory activities with IC_50_ values of 3.30 µg mL^−1^ and 5.62 µg mL^−1^, respectively (Figure 4). All fungal strains showed a moderate inhibitory effect on AChE activities, with IC_50_ values ranging from 36.61 to 118.87 µg mL^−1^. Out of four fungal strains, *Phyllosticta* sp. THG2-27 exhibited the most promising activity with an IC_50_ value of 36.61 µg mL^−1^, followed by *Phoma* sp. TKH3-2 with IC50 = 74.26 µg mL^−1^. On the contrary, the extracts from *E. sorghinum* THG01-18 and *C. siamense* THG1-17 showed lower inhibitory activity against AChE with 117.69 và 118.87 µg mL^−1^, respectively.

### 3.6. Growth Dynamics and Hup Biosynthesis Ability of Endophytic Fungi

**Figure 5:**
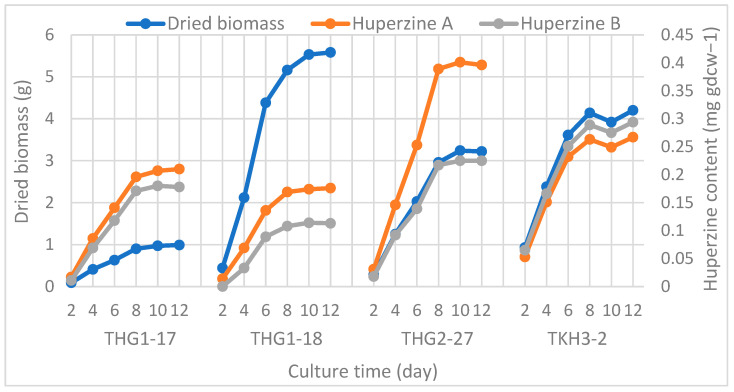
Growth dynamics and Hup biosynthesis of endophytic fungi.

To determine the growth time for the highest Hup yield of fungal strains for further research, four strains were studied growth dynamics when cultured on PDB medium for 2, 4, 6, 8, 10, and 12 days. At each culture period, the dried biomass of strains was collected to determine HupA and B yields (Figure 5).

The analysis revealed that Hup content was relatively proportional to the dry mass of fungal biomass. All strains reached the highest dry weight as well as Hup yield with stability between 8 and 12 days of culture (Figure 5). It shows that Hup synthesis depends on the growth of fungal cells during the culture stages. The fermentation time of 8 days was chosen for research on the genetic ability of Hup biosynthesis through cultivated generations of fungal strains.

### 3.7. Genetic Stability and Hup Yield of Endophytic Fungi

**Figure 6:**
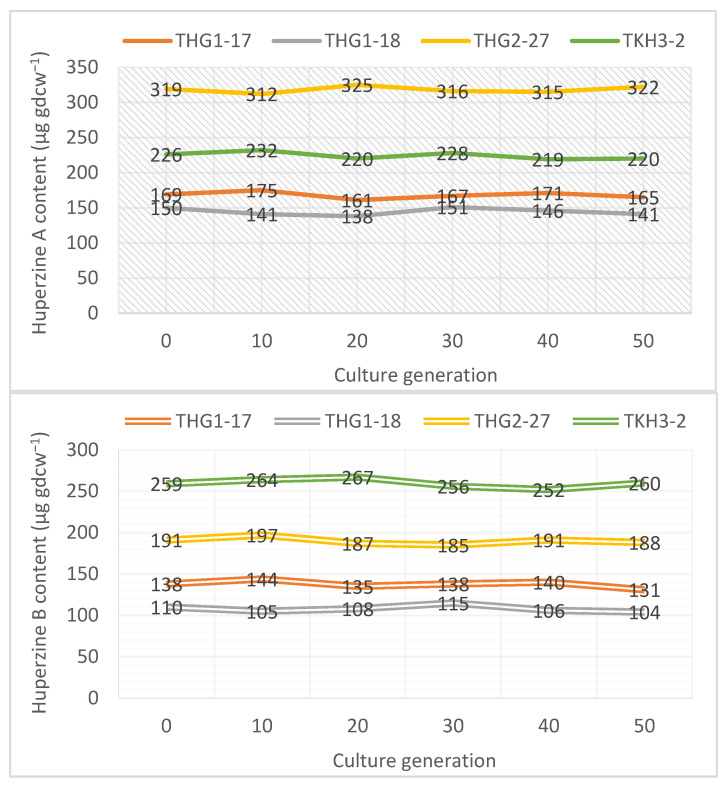
The ability to produce HupA and B of 4 wild strains (THG1-17, THG1-18, THG2-27, and TKH3-2) after 10th, 20th, 30th, 40th, and 50th generations.

The isolates cultured and preserved for 3 months of the 10th, 20th, 30th, 40^th^, and 50th generations of four wild strains (THG1-17, THG1-18, THG2-27, and TKH3-2) were determined for the ability to produce HupA and B. The Hup yield of the strains was quite stable between those generations and equivalent to the original generation. Although there were fluctuations, there were no significant differences in Hup content (Figure 6). This showed that genetic stability over generations of culture, as well as the strain storage period of 3 months, did not affect Hup biosynthesis.

## 4. Discussion

Dementia affects more than 50 million people worldwide, of which Alzheimer’s disease (AD) accounts for 50–75% of those people. It destroys brain cells and nerves by significantly disrupting neurotransmitters such as acetylcholine, which encounter significant disruption to their carrying of messages in the brain [1,2,3]. The use of AChE inhibitors is the approved pharmacotherapy approach to treat the symptoms of AD by improving the cholinergic deficit in the brain [4,7]. The current drugs used in the treatment of AD, such as tacrine, rivastigmine, galanthamine, and donepezil, have some significant side effects [8,9]. Therefore, the search for natural AChE inhibitors with higher efficacy and fewer side effects is necessary.

The alkaloids HupA and HupB originally extracted from *H. serrata* are known as highly selective, reversible, and potent inhibitors of AchE, of which HupA is widely used to treat AD worldwide while HupB is being researched as a precursor for developing novel multifunctional AChE inhibitors to treat AD [37,38,39,40]. Failures in chemical synthesis and in vitro culture have put *H. serrata* in danger of extinction and led to an extensive investigation of Hup from other *Lycopodiaceae* species. The endophytic fungi associated with *Lycopodiaceae* plants have the ability to produce HupA with high concentration. The microbial source of huperzine is available, and the hup-producing microorganisms can open up a promising approach for producing drugs containing huperzine to treat AD [27].

HupA has been determined in more than 50 *Lycopodiaceae* species from Australia, French Polynesia, Panama, Mexico, Malaysia, Poland, and, particularly, China, where *Lycopodiaceae* is distributed widely (*L. casuarinoides*, *H. carinata*, *H. squarrosa*, *H. selago, P. carinatus,* etc.). Most of these are at relatively low HupA concentrations, and the HupB concentration has been rarely quantified [6,15,16,17,18,19,20,21]. However, the HupA-producing endophytic fungus has received the most research attention in *H. seratta* with the presence of 12 genera, including *Acremonium*, *Shiraia*, *Cladosporium*, *Colletotrichum*, *Xylariales*, *Podospora*, *Fusarium*, *Trichoderma*, *Penicillium*, *Paecilomyces*, *Mucor*, *Alternaria* [27,28,29,30,31,32,33,34,35,41,42,43,44], and a few in other plant species such as *P. cryptomerianus* (*Blastomyces*, *Botrytis*) [45], *P. phlegmaria* (*Ceriporia, Hypoxylon*) [46], and *P. taxifolius* (*Fusarium*) [47]. Studies on endophytic fungi producing both Hup A and Hup B from the *Lycopodiaceae* have only been reported by Hu et al. (2018) with *Colletotrichum gloesporioides* (12.417 μg mL^−1^ HupA and 4.66 μg mL^−1^ HupB) isolated from *H. serrata* [48] (Table 4). In this study, the four endophytic fungi species producing both Hup A and Hup B were isolated from three *Lycopodiaceae* species, including strains *C. siamense* THG1-17 and *E. sorghinum* THG-1.18 (*P. squarrosus*), *Phyllosticta* sp. HG2-27 (*P. phlegmaria*), and *Phoma* sp. TKH3-2 (*L. clavatum*). This is the latest announcement of two plant species, *P. squarrosus* and *L. clavatum*, as novel sources of Hup-producing endophytic fungi. In addition, three endophytic fungal strains belonging to three genera, *Epicoccum*, *Phyllosticta*, and *Phoma*, as well as *Colletotrichum*, were novel hup-producing strains that generate both Hup A and HupB. This also demonstrates the diversity of Hup-producing endophytic fungi in *Lycopodiaceae* species as well as the diversity of host plant sources which they associate to produce identical or similar bioactive substances as host plants. Although the HupA content of the four strains were lower than that of previously reported by Han et al. (2020) [27] with HupA yields from 107.0 to 319.8 mg L^−1^, they were higher than many other reported strains (Table 4). In addition, they all have the ability to produce HupB with significant yield from 0.106 (THG1-18) to 0.263 (TKH3.2) mg gdcw^−1^. In order to utilize the strains in industrial production in the future, the genetic stability as well as the time of harvest of Hup need to be studied. In this work, four strains showed stability in Hup yield over 50 generations of culture with an in vitro storage period of 3 months and at a suitable Hup harvest time. Previous studies have only focused on HupA-producing fungal strains as a source of raw materials for the production of drugs to treat diseases. The isolated strains of HupB-producing fungi will provide a new source of raw materials for further research to develop drugs containing HupB for disease treatment in the future.

Vietnam is one of the potential countries for medicinal plants, especially those that are used to treat chronic diseases. Moreover, the development of natural medicinal raw materials for therapeutic purposes has been given more attention in recent years. More than 10 species of *Lycopodiaceae* have been found in the mountainous provinces of Vietnam, which are rare medicinal plants and are being preserved. HupA was found in *H. serrata* from the Lam Dong province of central Vietnam, with different HupA concentrations between the two samples collected in Spring (75.4 µg gdcw^−1^) and Autumn (92.5 µg gdcw^−1^) [49]. Our previous studies reported the presence of eight fungal genera, including *Alternaria*, *Fusarium*, *Trichoderma*, *Penicillium*, *Paecilomyces*, *Mucor*, *Acremonium*, and *Phoma*, inside *H. serrata*. Among them, two strains, *Penicillium* sp. LDL4.4 and *Fusarium* sp. Rsp5.2, were determined to produce HupA with yields of 0.168 and 0.01945 mg gdcw^−1^, respectively [33,34]. In this research, seven fungal genera (*Epicoccum*, *Schizophyllum*, *Aspergillus*, *Diaporthe*, *Curvularia*, *Colletotrichum*, and *Phyllosticta*) were found in three species of *Lycopodiaceae* that were not found in the *H. serrata*, showing the diversity of endophytic fungi in *Lycopodiaceae* species of Vietnamese.

In conclusion, the four fungal strains with potential yield of Hup A and B and high hereditary stability would provide a promising alternative resource for industrial-scale Hup production. However, to meet requirements for commercial HupA and HupB, strain improvement and optimization of the fermentation parameters will be the direction for our further research. Due to the novelty and potency of the findings, three patents describing HupA and B production by *Epicoccum sorghinum* THG1.18, *Phoma* sp. TKH3-2, and *Phyllosticta* sp. HG2-27 are under consideration by the Intellectual Property Office of Viet Nam under Application No. 2-2022-00056 (26 February 2022), No. 1-2022-02078 (20 May 2022), and No. 1-2022-02078 (2 June 2022), respectively.

## Figures and Tables

**Table 1:** Collected *Lycopodiaceae* species from the natural populations in Vietnam.

Plant Species * and Voucher Specimen Number	Location	Above Sea Level/m	HupA	HupB
*Lycopodium clavatum* L. DL-121120	Khanh Vinh, Khanh Hoa province		+	+
	(12°21′35′′ N 108°46′34′′ E)	775		
*Phlegmariurus phlegmaria* (L.) T.Sen & U.Sen. DL-151120	Quan Ba, Ha Giang province		+	+
	(23°05′15′′ N 104°51′00′′ E)	1459		
*Phlegmariurus squarrosus* (G.Forst.) Á.Löve & D.Löve. DL-161120	Quan Ba, Ha Giang province		+	+
	(23°05′32′′ N 104°56′40′′ E)	1068		
	Khanh Vinh, Khanh Hoa province		+	+
	(12°24′01′′ N 108°44′54′′ E)	1103		

* *Phlegmariurus phlegmaria* = *Lycopodium phlegmaria*, *Phlegmariurus squarrosus* = *Lycopodium squarrosus* = *Huperzia squarrosa* [18,26,27].

**Table 2:** The content of Hup in fungal strains.

Strain	HupA/L Liquid Culture (mg L^−1^)	HupA/g Dried Mycelium (mg gdcw^−1^)	HupB/L Liquid Culture (mg L^−1^)	HupB/g Dried Mycelium (mg gdcw^−1^)
THG1-17	0.585	0.174	0.437	0.141
THG1-18	0.758	0.147	0.548	0.106
THG2-27	1.366	0.324	0.582	0.197
TKH3-2	0.703	0.221	0.837	0.263

**Table 3:** Identification of the Hup-producing fungal strains.

Strain	Plant Organ	Size of ITS Gene (bp)	GenBank Accession No.	Closest Match	Query Coverage (%)	Percentage Identity (%)
THG1-17	Stem	523	OM943918	*Colletotrichum siamense* (MW322808, QQ652481)	100	99.43
THG1-18	Stem	488	OM943947	*Epicoccum sorghinum* (MN006431, KX758542)	99	99.18
THG2-27	Stem	602	ON024637	*Phyllosticta capitalensis* (MT085755, MN744737)	93	99.29
TKH3-2	Stem	482	OM943953	*Phoma* sp. (MT557514, MT557442)	94	99.78

**Table 4:** The Hup-producing endophytic fungi isolated *Lycopodiaceae* species.

Fungi	Host Plant	HupA Yield	HupB Yield	References
*Acremonium* sp. 2F09P03B	*Huperzia serrata*	8.32 µg L^−1^	none	Li et al. (2007) [41]
*Shiraia* sp. Slf14	*H. serrata*	327.8 µg L^−1^ (142.6 µg gdcw^−1^)	none	Zhu et al. (2010) [35]
*Cladosporium cladosporioides* LF70	*H. serrata*	56.84 µg L^−1^ (39.61 µg gdcw^−1^)	none	Zhang et al. (2011) [28]
*Colletotrichum gloeosporoides* ES026	*H. serrata*	30 µg gdcw^−1^	none	Zhao et al. (2011) [42]
*Xylariales* sp. SY-02	*H. serrata*	26,4 µg L^−1^	none	Su et al. (2011) [43]
*Podospora* sp. S29	*H. serrata*	50.6 µg gdcw^−1^	none	Dong et al. (2014) [29]
*Trichoderma harzianum* L44	*H. serrata*	37.63 µg gdcw^−1^	none	Dong et al. (2014) [29]
*Paecilomyces tenuis* YS-13	*H. serrata*	21 µg L^−1^	none	Su and Yang (2014) [30]
*Ceriporia renrata* MY311	*Phlegmariurus plegmaria*	40.53 µg L^−1^	none	Zhang et al. (2015) [46]
*Hypoxylon investiens* NX9	*P. plegmaria*	27.48 µg L^−1^	none	Zhang et al. (2015) [46]
*Fusarium oxysporum* SNZ-12	*H. serrata*	11.1 mg L^−1^	none	Hong-quin et al. (2019) [44]
*Colletotrichum gloesporioides*	*H. serrata*	12.417 μg mL^−1^	4.66 μg mL^−1^	Hu et al. (2018) [48]
*Alternaria brassicae* AGF041	*H. serrata*	42.89 µg gdcw^−1^	none	Zaki et al. (2019) [31]
*Penicillium* sp. LDL4.4	*H. serrata*	1.38 mg L^−1^ (168.9 µg gdcw^−1^)	none	Le et al. (2019) [33]
*Fusarium* sp. Rsp5.2	*H. serrata*	19.45 μg gdcw^−1^	none	Le et al. (2020) [34]
*Mucor racemosus* NSH-D	*H. serrata*	107.0 mg L^−1^	none	Han et al. (2020) [27]
*Mucor fragilis* NSY-1	*H. serrata*	181.7 mg L^−1^	none	Han et al. (2020) [27]
*Fusarium verticillioides* NSH-5	*H. serrata*	117.1 mg L^−1^	none	Han et al. (2020) [27]
*Fusarium oxysporum* NSG-1,	*H. serrata*	111.1 mg L^−1^	none	Han et al. (2020) [27]
*Trichoderma harzianum* NSW-V	*H. serrate*	319.8 mg L^−1^	none	Han et al. (2020) [27]
*Fusarium* sp. C17	*P. taxifolius*	3.2 µg gdcw^−1^	none	Cruz-Miranda et al. (2020) [47]
*Colletotrichum boninense* HS7-1	*H. serrata*	3.7 µg gdcw^−1^	none	Cui et al. (2021) [32]
*Colletotrichum siamense* THG1-17	*P. squarrosus*	0.174 mg gdcw^−1^	0.141 mg gdcw^−1^	Present study
*Epicoccum sorghinum* THG-1.18	*P. squarrosus*	0.147 mg gdcw^−1^	0.106 mg gdcw^−1^	Present study
*Phyllosticta* sp. HG2-27	*P. phlegmaria*	0.324 mg gdcw^−1^	0.197 mg gdcw^−1^	Present study
*Phoma* sp. TKH3-2	*Lycopodium clavatum*	0.034 mg gdcw^−1^	0.028 mg gdcw^−1^	Present study

## Data Availability

The datasets analyzed in this study are available from the corresponding author upon reasonable request.
